# Performance Monitoring of Anaerobic Digestion at Various Organic Loading Rates of Commercial Malaysian Food Waste

**DOI:** 10.3389/fbioe.2022.775676

**Published:** 2022-03-24

**Authors:** Afifi Zainal, Razif Harun, Syazwani Idrus

**Affiliations:** ^1^ Department of Generation and Environment, Renewable Energy and Green Technology Unit, TNB Research Sdn. Bhd., Kajang, Malaysia; ^2^ Department of Chemical and Environmental Engineering, Faculty of Engineering, Universiti Putra Malaysia, Serdang, Malaysia; ^3^ Department of Civil Engineering, Faculty of Engineering, Universiti Putra Malaysia, Serdang, Malaysia

**Keywords:** anaerobic digestion (AD), organic fraction of municipal solid waste (OFMSW), continuous stirred-tank reactor (CSTR), biomethane potential (BMP) test, organic loading rate (OLR)

## Abstract

Application of anaerobic digestion (AD) has become common in treating palm oil mill effluent in Malaysia; however, employing AD in treating the organic fraction of municipal solid waste (OFMSW), especially food waste, is still scarce. This study aims to characterize the commercial Malaysian food waste (CMFW) and determine its potential as sustainable bioenergy feedstock through biogas production. The sample was digested *via* the biomethane potential (BMP) test with the variation of organic loading rates (OLRs), ranging from 0.38 to 3.83 gCOD/L. day, under mesophilic conditions. The digestion process was further evaluated in continuous operation using a 6-L continuous stirred-tank reactor (CSTR). The kinetic properties of the process were also determined. It was found that the CMFW had a significant amount of chemical oxygen demand of 230 g/L and an acidic pH of 4.5 with the carbon to nitrogen (C/N) ratio at 121:1. A maximum methane composition of 81% was obtained at 1.92 gCOD/L in the BMP test with specific methane production (SMP) at 0.952 L. CH_4_/L.COD fed. The biogas production was well-fitted with the modified Gompertz model with *R* ^2^ at 0.9983 and the maximum biogas potential production rate at R_m_ 0.1573 L/day, whereas in the CSTR operation, a maximum methane composition of 85% was produced at OLR 6 gCOD/L. day with the SMP of 1.13 L. CH_4_/L.COD fed. The CSTR system was in high stability as the pH was maintained in a range of 6.6–6.7, with an alkalinity ratio of 0.28. This study indicates the CMFW is a sustainable feedstock for biogas production in Malaysia. Toward a circular economy approach, the authorities shall introduce commercial scale CMFW AD as part of managing municipal solid waste issues in Malaysia.

## 1 Introduction

The World Bank estimated 2.01 billion of municipal solid waste (MSW) is generated globally, with East Asia and the Pacific producing the highest at a staggering 468 million tons per annum or equivalent to 22% waste generated (Kaza et al., 2018). In Malaysia, it is reported that around 13.87 million tons of solid waste was generated annually (SWCorp 2019). A report by the National Solid Waste Management Department of Malaysia stated that the major fraction of solid waste was contributed by food waste (FW) with 44.5% out of the overall composition. This percentage corresponds to the global waste composition, where 44% of waste comes from food or green waste (JPSPN 2013; Kaza et al., 2018; SWCorp 2019).

As a result of rapid population growth and urbanization, Malaysia’s annual waste generation is increasing at an alarming rate. Issues on solid waste are getting more crucial and challenging in Malaysia as currently, it depends on the conventional landfill instead of implementing a sustainable approach. Also, solid waste management is a complex issue in Malaysia as it involves various stakeholders. It requires a cumulative effort that includes solid waste generation, storage, collection, transport, processing, and disposal acts that should also favor the environmental, economic, and public concerns (Razali et al., 2019). Although a series of actions have been taken in managing solid waste by the Malaysian authority, it is still far behind developed countries (Razali et al., 2019). Although solid waste has been segregated at sources, only a few components of these segregated materials such as paper, plastic, glass, and aluminum can be recycled. The major portion of this segregated solid waste, which is food waste, remains mixed again with other waste streams when treated at the conventional landfill. This is detrimental to the effort for the separation at source initiative, as stated in the regulation that has been enforced in Malaysia.

There are several technologies for treating municipal and food wastes. Landfilling has been the main treatment as it is the cheapest management option (Galanakis 2015). Several other options include biofuel conversion through the biochemical pathway (i.e., anaerobic digestion and bioethanol) (Dahiya et al., 2018) or thermochemical treatment (i.e., incineration, pyrolysis, and gasification), composting, and valorization of food waste (Galanakis, 2015; Imbert 2017; Xu et al., 2018). Since the landfilling, composting, and incineration technologies bring a negative impact to the environment, AD has been proposed as a relatively cost-effective technology for treating waste and renewable energy production (Xu et al., 2018). The versatility of the anaerobic digestion technology in treating multiple types of organic waste, including the OFMSW and in parallel generate renewable energy, has led to a greater interest for its application. Through biotransformation and the energy recovery approach, AD turns organic waste into energy-rich biogas, which can be utilized as a renewable energy feedstock to generate heat and electricity. In parallel, AD produces a nutrient-rich fertilizer that can be applied for landscaping, nursery, or the agriculture industry (Tyagi et al., 2018). As the volume of food waste generation is high in Malaysia, the OFMSW anaerobic digestion is deemed an available and promising technology to utilize the OFMSW as feedstock material for renewable energy. This approach could uplift the waste management a circular economy, thus bringing a positive impact to the environment, economy, and society.

In particular, researchers have extensively conducted research on food waste as a substrate of anaerobic digestion. This is because food waste has a high potential in producing biogas either in mono-digestion or co-digestion with other organic sources. A review by Bong et al., 2018 found that the study on food waste as biogas feedstock is highly concentrated in countries such as China and Japan and a few other countries such as Korea, Pakistan, Mexico, the United Kingdom, and Ireland (Bong et al., 2018). Although utilizing food waste as feedstock for biogas production has been studied to be commercially available in many countries, it is not the case in Malaysia. Albeit having difficulties in sustainably managing this solid waste, ironically, the study on food waste as feedstock for biogas production in Malaysia is still scarce (Ghafar 2017).

In addition, the differences of food waste variation characteristics among different countries and cities for anaerobic digestion have been elucidated by Bong et al., 2018. They concluded that the effective utilization of this technology would, however, require further studies on the variation of the quantity and quality of the available FW, as well as the suitability between the variation of the characteristics and its respective improvement method. Therefore, this study evaluates the commercial Malaysian food waste characteristic and its suitability for biogas feedstock by conducting the biomethane potential test and evaluating its digestion performance in a continuous stirred-tank reactor.

## 2 Materials and Methods

### 2.1 Feedstock and Inoculum Characteristics

The CMFW sample was collected from the Malaysian food restaurants located at Precinct 15, Putrajaya, Malaysia. The CMFW consisted of a portion of leftover cooked foods, especially rice, and side dishes. Meat (chicken), fish bones, and eggshells were segregated, ground, and mixed using a food processor before the experiments. Meanwhile, the inoculum used in this experiment was sourced from a wastewater treatment plant located at the Faculty of Engineering, Universiti Putra Malaysia (UPM), Malaysia. Scanning Electron Microscopy (SEM)/Energy-dispersive X-ray (EDX) spectroscopy was employed for inoculum description.

### 2.2 Biomethane Potential Test

The BMP test was performed in a series of scotch bottles operating as a batch anaerobic digester. The organic loading rate (OLR) was designed based on the inoculum to food waste (I/F) ratio of 300:1, 150:1, 100:1, 75:1, 60:1, and 30:1, as illustrated in Table 1. Each of the digesters was added with an equal amount of 450 ml of inoculum, and the CMFW was added according to the calculated OLR. The digesters were then filled up with distilled water up to 900 ml, leaving 100 ml for the biogas headspace. All the digesters were flushed with 100% nitrogen gas (N_2_) for 2 mins to avoid the presence of air in the headspace before being sealed with a rubber stopper. The digester was incubated in a water bath at 38 ± 2°C. Each of the digesters was shaken every day for 1 min in a water bath. The biogas production was observed by the water displacement method using a graduated measuring cylinder. The methane content was analyzed by gas chromatography (GC) and calculated by subtracting the corresponding values for control and substrate runs. The physicochemical parameters such as the chemical oxygen demand (COD), total suspended solid (TSS), volatile suspended solid (VSS), and ammonia–nitrogen (NH_3_-N) were measured before and after the BMP test. Table 1 shows the experimental setup for the BMP test, respectively.

**TABLE 1:** Experimental setup for the BMP test.

Samples	I/F ratio	Vol. of CMFW fed (ml)	OLR (gCOD/L.day)	S, influent (g.COD/L)
Blank	-	0.0	-	-
A	300:1	1.5	0.38	0.77
B	150:1	3.0	0.77	1.53
C	100:1	4.5	1.15	2.30
D	75:1	6.0	1.53	3.07
E	60:1	7.5	1.92	3.83
F	30:1	15.0	3.83	7.67

Digester working volume = 900 ml.

Inoculum volume = 450 ml.

Total COD = 230 g/L.

### 2.3 Continuous Anaerobic Digestion System

Continuous anaerobic digestion was conducted using a laboratory-scale continuous stirred-tank reactor (CSTR). The reactor was subjected to a mesophilic condition at 38 ± 2°C. The experiment was carried out in a 6-L CSTR with 5 L of working volume: 3 L for inoculum and 2 L for the substrate. The organic loading rate (OLR) was manipulated, and its biogas and methane production was evaluated. The CMFW was fed to the digester at different OLRs ranging from 1.3 to 8 g COD/L. day in the continuous mode using a constant hydraulic retention time (24 h). Change in the feed concentration from one OLR to another was performed once the system reached a steady state with an insignificant difference in biogas production for three to four consecutive days (Choi et al., 2013). The performance evaluation parameters for the AD process, such as COD removal, VS destruction, alkalinity, ammonia–nitrogen, and biogas methane yield, were evaluated during the 85 days of the AD process.

### 2.4 Analytical Methods

#### 2.4.1 Operating and Yield Parameters

The operating and yield parameters of the anaerobic process throughout this study were followed (Mata-Alvarez and S. Mace 2007; Khanal 2008; Khanal and Li 2017), while the specific methane production (SMP) and methane production rate (MPR) were an adaptation from the specific gas production (SGP) and gas production rate (GPR) (Mata-Alvarez and S. Mace 2007).

#### 2.4.2 Biomethane and Gas Analysis

The biogas production of the CSTR reactor was monitored and recorded daily using the water displacement method. The biogas will then be collected in a Tedlar bag, and the biogas composition was analyzed using a gas chromatograph (HP 6890 N) (Agilent, Santa Clara, CA 95051, United States) equipped with a thermal conductivity detector (TCD) (Agilent, Santa Clara, CA 95051, United States). The column used was HP Molesieve (Agilent Technologies, Santa Clara, CA, United States) of 30 m length × 0.5 mm ID × 40 μm film thickness capillary column. The splitless inlet, oven, and TCD detector temperatures will be kept at 60°C, 70°C, and 200°C. Argon was used as the carrier gas, while nitrogen was used as the makeup gas.

#### 2.4.3 Physicochemical Analysis

The chemical oxygen demand (COD), total solid (TS), volatile solid (VS), and alkalinity were measured following standard methods for the examination of water and wastewater by the American Public Health Association (APHA, 2017). pH was tested using the pH Meter Mettler-Toledo AG, 8603 Schwerzenbach, Switzerland. Oil and grease (O&G) was analyzed using the partition gravimetric method. DR 900 multi-parameter portal calorimeter was used to analyze total nitrogen (TN), phosphate (PO_4_^3-^), and ammonia–nitrogen (NH_3_-N). Microwave digestion inductively coupled plasma–mass spectrometry (ICP-MS) was employed for trace element analysis. Simple sugar was analyzed through high-performance liquid chromatography–evaporative light-scattering detector (HPLC-ELCD) method. Analysis of volatile fatty acid (VFA) was performed using an Agilent 1200 HPLC system, United States, with the Rezex ROA column Phenomenex, United States, and equipped with a refractive index detector (RID).

#### 2.4.4 Kinetic Study

A kinetic study can give an insight into the effect of fed to inoculum on biogas production. In this study, a modified Gompertz model was chosen to describe the behavior of the anaerobic process of the CMFW in producing biogas. Through cumulative biogas yield, M, we obtained the following parameters which were determined through Microsoft Excel 2016 solver analysis tools

$$M=P\exp\left\{-\exp\left[\frac{R_{m}e}{P}\left(\lambda -t\right)+1\right]\right\}$$

where *P* (L) is the biogas yield potential, *R*_*m*_ (L/day) is the maximum biogas production rate, and *λ* (day) is the lag phase, with *e* being the exponential constant of 2.7183 (Xie et al., 2016; Li Y. et al., 2018; Silva et al., 2018).

## 3 Result and Discussion

### 3.1 Characterization of the Substrate and Inoculum

The characteristic of the CMFW substrate is presented in Table 2. It was found that the TCOD concentration of the CMFW was 230 g/L. The obtained value was three times less than the TCOD reported by Chua et al., 2013. The SCOD of this sample was 149 g/L, indicating 64% of the TCOD was in a soluble form. A high concentration of the SCOD indicates the CMFW can be easily digestible, hence advantageous to be used as a feedstock for the AD process. Also, the pH for the CMFW was at 4.5, and this pH was slightly lower than the reported literature, where the average pH of food waste is around 5.1 (Tampio et al., 2014; Yeshanew et al., 2016). In terms of bromatological characteristic, the CMFW consists of carbohydrates, protein, and fat, on average (%) 72.1, 24.1, and 3.8, respectively, whereas the typical food waste fraction has the composition of carbohydrates 41–62%, proteins 15–25%, and lipids 13–30% (Braguglia et al., 2018). This indicated that the CMFW used in this study had a slightly different composition from the typical food waste as it is high in carbohydrates and low in lipids.

**TABLE 2:** Characteristic of commercial Malaysian food waste.

Parameter	Result	Unit
Physicochemical characteristics		
Total chemical oxygen demand (TCOD)	230	g/L
Soluble chemical oxygen demand (SCOD)	149	g/L
Volatile solid (VS.)	0.048	g/L
Total solid (TS)	0.725	g/L
VS./TS	6.621	%
Total nitrogen (TN)	1.89	g/L
Ammonia–nitrogen (AN)	0.213	g/L
C/N ratio	121:1	
Oil & grease	16	mg/L
pH	4.5	
Bromatological characteristics		
Energy	131	kcal/100 g
Total carbohydrate^b^	0.0	g/100 g
Protein	24.1	g/100 g
Total fat	3.8	g/100 g
Moisture	70.5	g/100 g
Ash	1.6	g/100 g
Fructose	0.23	g/100 g
Glucose	N. D. (<0.001)	g/100 g
Sucrose	N. D. (<0.001)	g/100 g
Maltose	1.6	g/100 g
Trace Elements		
Phosphorus, P	69.133	g/L
Sodium, Na	3.236	g/L
Potassium, K	0.985	g/L
Calcium, Ca	0.692	g/L
Magnesium, Mg	0.155	g/L
Iron, Fe	13.7	mg/L
Zinc, Zn	3.81	mg/L
Chromium, Cr	2.03	mg/L
Manganese, Mn	1.7	mg/L
Selenium, Se	1.5	mg/L
Antimony, Sb	0.304	mg/L
Mercury, Hg	0.199	mg/L
Arsenic, As	0.192	mg/L
Lead, Pb	0.022	mg/L
Cadmium, Cd	0.011	mg/L
Tin, Sn	N.D.	mg/L

N.D. = Non-detected.

a C/N ratio is derived from the TCOD and T.N. value.

b Total carbohydrate = 100–(%Ash + %Moisture + %Protein + %Fat).

On the other hand, the carbon to nitrogen (C/N) ratio for the CMFW was high at 121. A well-balanced feed with sustainable digestion requires a C/N ratio of 25–30, as reported by (Zhang et al., 2014). However, some of the researchers had a C/N ratio of food waste in a range of 13.2–24.5 (Braguglia et al., 2018) or below then 44 (Zhang et al., 2014; Deepanraj et al., 2017). Therefore, this CMFW has a unique characteristic that is high in organic carbon as compared to other sources of food waste. Understandably, Malaysian uptake on carbohydrates, especially rice as staple food has been the bulk of food waste. In tackling this issue, the introduction of co-substrate with higher nitrogen content can be put forward in balancing the C/N of the CMFW for enhancing the process stability. A recent study performed by Latha et al. (2019) had shown the improvement of biogas production with *in situ* 88% methane enrichment when mixing food waste with sewage sludge or with cattle manure for the digestion process. This strategy could balance the nutrients and reduce the accumulation of the VFA in the digestate. Over time, these strategies have been consistently showing a synergistic effect in biogas production (Arelli et al., 2018; Tong et al., 2019).

Parts of organic carbon, nitrogen, and phosphorus are macronutrients essential for the AD process to run in optimal conditions (Khanal 2008). The nitrogen and phosphorus contained in the CMFW were 1.89 g/L and phosphorus 69.13 g/L, respectively. Thus, the CMFW carbon to nitrogen to phosphorus (C:N:P) ratio was 600:5:180, which indicates the substrate is slightly less in nitrogen but high in phosphorus as per the benchmark with the ideal ratio at 600:7:1 (Mata-Alvarez and S. Mace, 2007). On the other hand, it was found that the CMFW was packed with essential micronutrients: sodium, calcium, magnesium, iron, zinc, and selenium (Table 2). The presence of micronutrients in small quantities can stimulate bacterial growth and microorganism activities; however, it can be inhibitory if it surpassed a certain level (Mata-Alvarez and S. Mace, 2007; Braguglia et al., 2018). Menon et al’s., 2017 study shows that supplements of micronutrients such as magnesium, calcium, cobalt, and nickel will enhance 50% biogas productivity and reduced processing time.

**FIGURE 1:**
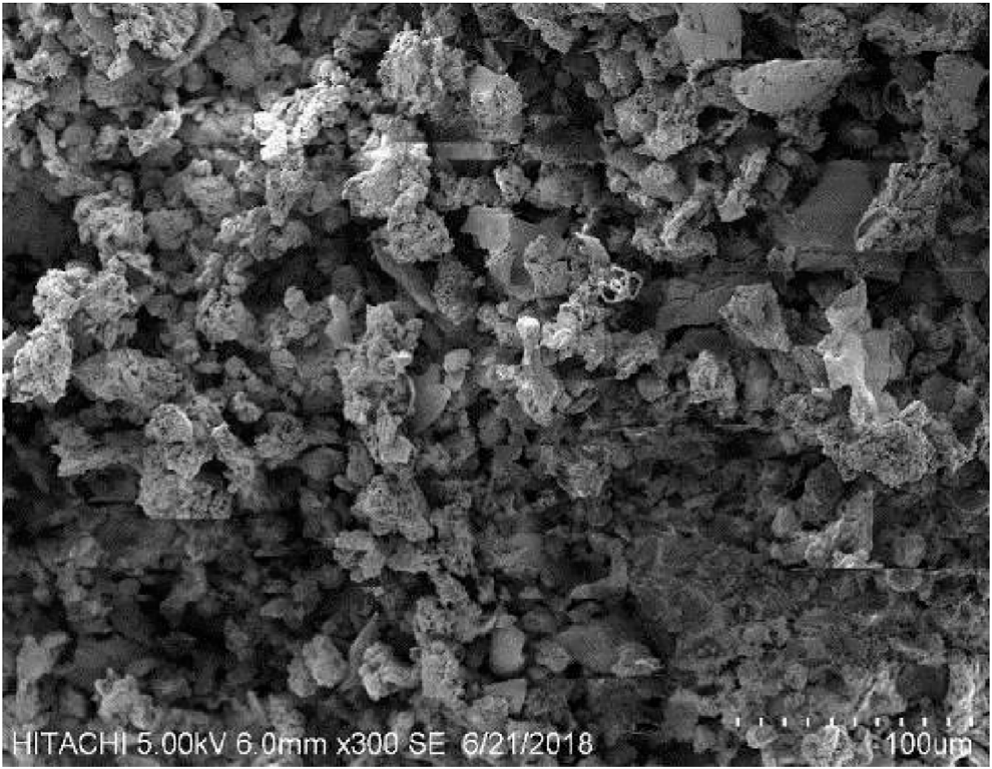
Scanning Electron Microscopy (SEM) images of inoculum.

In terms of inoculum description, Figure 1 shows the SEM images of the inoculum sourced from a wastewater treatment plant which contained a fine particle in a black mixture with relatively small-size granules.

The raw COD, total solids, and volatile solid of the inoculum were 21 g/L, 223.13 mg/L, and 75.1 mg/L, respectively. EDX spectrum elements of the inoculum sample are shown in Table 3. It was found that the inoculum majorly consists of carbon (C) and oxygen (O) atoms with 48.07 and 18.67%, respectively. In general, no harmful elements were found in the inoculum, hence making it suitable to be used in the digestion process. Moestedt et al. (2020) found that the inoculum sourced from the wastewater treatment plant for the anaerobic digestion process will produce higher acetate and lower lactate, when treating food waste co-digested with sewage sludge (Moestedt et al., 2020). In comparison to elements found in this study, previous characteristic of the wastewater treatment inoculum shows that it also contained calcium, iron, molybdenum, aluminum, and phosphorus. The study found that the presence of molybdenum and nickel in the inoculum will enhance methane production and shorten the lag-phase food waste anaerobic digestion (Parra-Orobio et al., 2018).

**TABLE 3:** Inoculum description.

EDX Spectrum Elements	Result	Unit
Carbon, C	48.07	%
Oxygen, O	18.67	%
Aluminum, Al	2.81	%
Silicon, Si	6.28	%
Phosphorus, P	2.37	%
Calcium, Ca	5.70	%
Iron, Fe	9.88	%
Molybdenum, Mo	6.22	%
Total	100.00	%

### 3.2 Commercial Malaysian Food Waste Biomethane Potential Test

**FIGURE 2:**
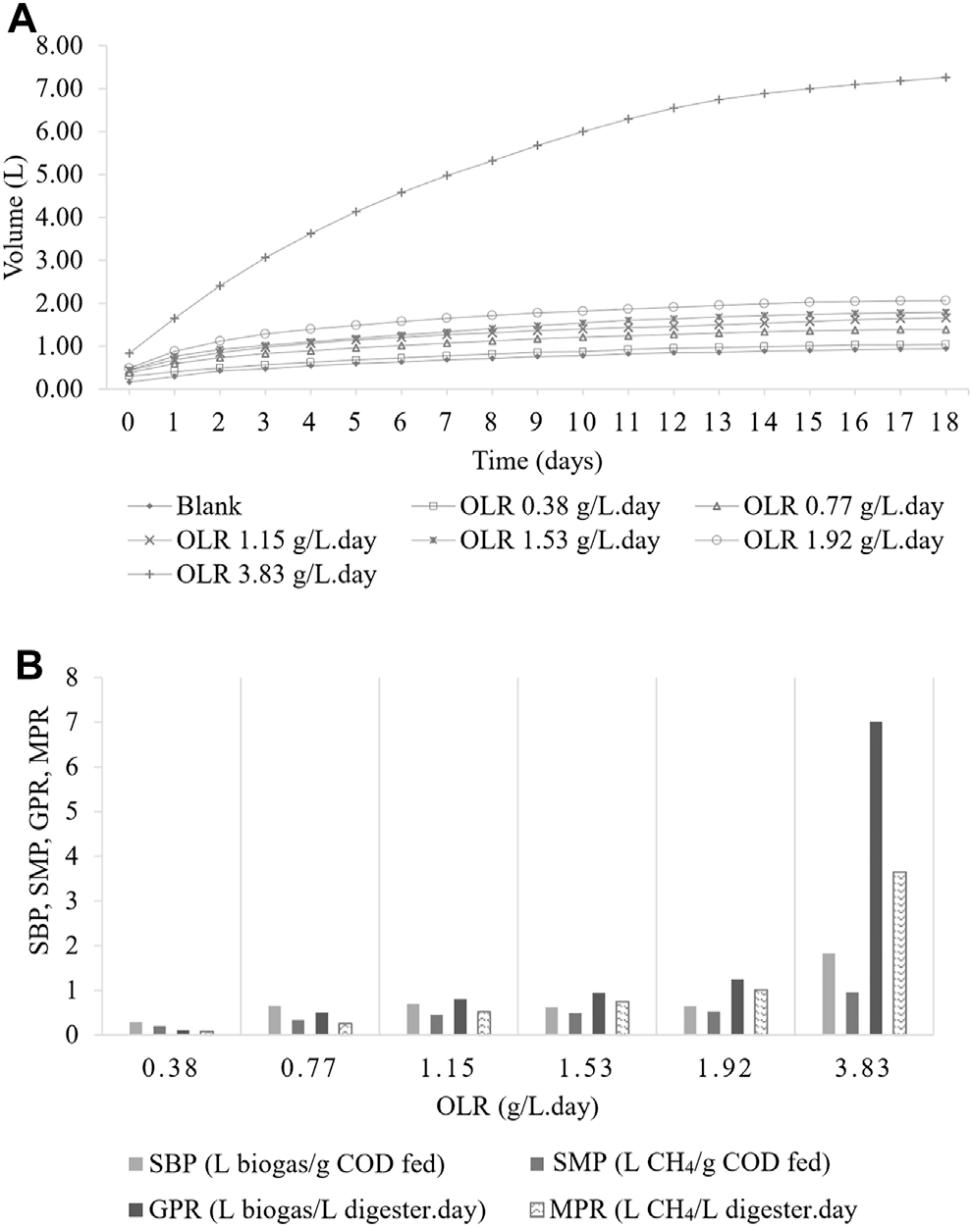
Biomethane Production (BMP) Test. **(A)** Cumulative biogas production. **(B)** Biogas productivity.

The BMP test was carried out to determine the degradability of the CMFW. Figure 2A shows the profile of biogas production from six different OLRs: 0.38, 0.77, 1.15, 1.53, 1.92, and 3.83 gCOD/L. day.

It was noted that the blank sample was 100% inoculum run in parallel with the BMP experiments and used as the control. As shown in Figure 2A, the average total cumulative biogas produced were 1.04, 1.39, 1.66, 1.79, 2.06, 7.26 L, and 0.94 L for OLRs 0.38, 0.77, 1.15, 1.53, 1.92, and 3.83 gCOD/L. day and a blank sample. After deducting the amount of the biogas produced in the blank sample, Table 4 shows the amount of biogas produced by the feeds were 0.10, 0.45, 0.72, 0.85, 1.12, and 6.32 L, respectively.

**TABLE 4:** Biogas production and methane composition.

OLR (gCOD/L.day)	Vol. Biogas (L)	Vol. CH_4_ (L)	CH_4_ percentage (%)
0.38	0.10	0.07	70
0.77	0.45	0.23	51
1.15	0.72	0.47	65
1.53	0.85	0.67	79
1.92	1.12	0.91	81
3.83	6.32	3.29	52

In terms of methane composition, from Table 4, the OLR 1.92 gCOD/L. day had the highest percentage of 81%, whereas OLR 0.77 gCOD/L. day had the lowest percentage of 51%. It can be observed that there was a minimal step increment of biogas and methane yield when increasing the OLRs. The graph also showed that the yield of methane was reasonably stable within the same OLR until the OLR was changed. However, as the OLR reached 3.83 gCOD/L. day, the yield of biogas and methane was sharply reduced with nearly 6 and 4 folds compared to OLR 1.92 gCOD/L. day. This was due to the imbalance of production and consumption of VFAs as food waste is known as a rapid biodegradable organic (Braguglia et al., 2018; Pramanik et al., 2019).

Four indicators which are the specific biogas production (SBP), specific methane production (SMP), gas production rate (GPR), and methane production rate (MPR) were used to measure the CMFW biogas productivity. As shown in Figure 2B, it can be observed that at the low OLRs (0.77–1.92 gCOD/L. day), there were no significant changes of the SBP (0.696–0.616 L biogas/g COD fed). In contrast, the SMP, GPR, and MPR were increased as the OLR increased. However, at a higher OLR of 3.83 gCOD/L. day, biogas productivity trends changed with the SBP, SMP, GPR, and MPR, abruptly reaching at 1.831 L biogas/gCOD fed, 0.952 L CH_4_/gCOD fed, 7.013 L biogas/L digester. day, and 3.647 L CH_4_/L digester. day. In addition, the 3.83 gCOD/L. day OLR produced more biogas but with less methane; these indicated that methanogens had been affected by the increase of the organic matter in the CMFW, the SBP, and GPR, significantly two folds higher than the SMP and MPR. A higher concentration of organics in the CMFW leads to the rapid degradation of food waste. Thus, the anaerobic microorganism has outperforming methanogens archaea, a methane-producing microorganism (Khanal and Li 2017). The rapid degradation process of food waste produced long-chain VFAs and less acetic acid, thus lowering the production’s methane concentration.

### 3.3 Monitoring Parameters in the Biomethane Potential Test

It was observed the COD removal’s efficiencies for the CMFW sample were 86.8, 95, 96.7, 97.6, 98.1, and 97.7% for OLRs 0.38, 0.77, 1.15, 1.53, 1.92, and 3.83 gCOD/L. day, as shown in Table 5, respectively. These removal efficiencies indicated that the bacterial activity was adapted to the subjected environment. The highest COD removal of 86% was achieved at the OLR 0.38 with 0.38 g COD/L. day OLR. This value still can be considered high for the cumulative biogas produced by the reactor and within the limit of acceptance. In the subsequent reactors, more than 90% of COD removal efficiencies were achieved.

**TABLE 5:** Monitoring parameters in the BMP test.

OLR (gCOD/L.day)	COD removal (%)	Ammonia Nitrogen (mg/L)	pH	IA/PA
0.38	86.83	183.75	7.05	0.14
0.77	94.90	172.50	6.98	0.23
1.15	96.65	192.50	7.01	0.28
1.53	97.59	180.00	6.97	0.24
1.92	98.07	187.50	6.99	0.23
3.83	97.72	272.50	7.14	0.53

The values of NH3-N at OLRs 0.38, 0.77, 1.15, 1.53, 1.92, and 3.83 gCOD/L. day were 183.75, 172.5, 192.5, 180, 187.5, and 272.5 mg/L, respectively. It was observed that the NH3-N values for each sample were in a range of 172.5–192.5 mg/L except for the OLR 3.83, where it contained a higher NH3-N of 272.5 mg/L. It is noted that none of the values obtained at each sample exceeded the limit to which inhibition can occur, as stated previously. Ammonia is generated by the biodegradation of nitrogenous compounds, mostly in the form of proteins. Generally, for a high-rate anaerobic digester, concentration 1700–1800 mg/L would lead to digester failure. For a normal digester, a concentration of 3,000 mg/L will lead to partial inhibition. However, a study conducted by Yenigün and Demirel 2013 showed that acclimatized archaea can stand up to 5,000 mg/L of NH3-N. Ammonia inhibition includes an increase of the maintenance energy requirement, a change in the intracellular pH, and inhibition of a specific enzyme reaction (Akindele and Sartaj 2018).

Indication of digester stability can be made through the measurement of pH and the intermediate alkalinity and partial alkalinity ratio (IA/PA). It was found that all the samples had a stable pH, which was at a neutral range (pH 6.97–7.14). The anaerobic digestion process occurs at a pH of 6.0–8.3, while most methanogens have an optimum pH between 7 and 8, while the acid-forming bacteria are often at a lower range (Angelidaki and Sanders 2004). Meanwhile, alkalinity and pH are related to each other as alkalinity acted as a buffering agent in controlling acidity derived from acidogenesis in the anaerobic treatment process (Tabatabaei et al., 2011). According to Table 5, it can be deduced that all other reactors were able to maintain a stable operation condition, as revealed by the results range 0.14–0.23, except at the highest OLR 3.83, where the alkalinity ratio spiked off at 0.53. The alkalinity ratio below 0.3 shows the system in a stable condition (Li L. et al., 2018).

In contrast, a higher ratio than 0.3 indicates volatile fatty acid accumulation, which leads to a low percentage of methane production. These can be seen in the sample for OLRs 1.92 and 3.83 as the OLR had a higher alkalinity ratio which increased from 0.23 to 0.53. This resulted in reducing the methane percentage from 81 to 53%, as shown in Table 4. Therefore, whenever a value of less than 0.3, it is an indication that even if VFA accumulation occurred, it was controlled by the digester’s buffer capacity.

### 3.4 Continuous Anaerobic Digestion System

#### 3.4.1 Commercial Malaysian Food Waste Digestion Performance in a Continuous Stirred-Tank Reactor

**FIGURE 3:**
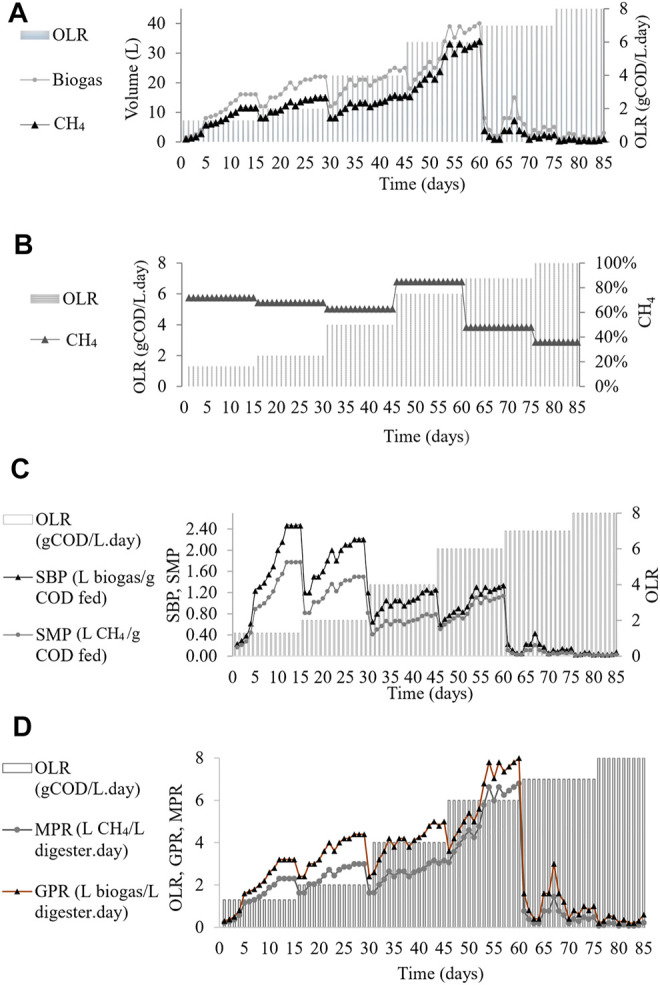
CMFW digestion performance in CSTR. **(A)** Biogas and methane yield. **(B)** Methane composition. **(C)** Specific biogas and methane production. **(D)** Biogas and methane production rate.

The daily amount of biogas and methane yield at various OLRs during the CSTR operation period is shown in Figure 3A. The changes of OLRs corresponding to the changes in the influent COD concentration were performed from one level to another once the biogas production achieved a steady-state condition with less than 5% variation (Poh and Chong 2014). The maximum volume of biogas production of the reactor was 40 L and found at OLR 6 gCOD/L. day, and therefore, this represents the optimum OLR.

This study found that the increase in the organic loading rate led to an increase in biogas production at a 1-day hydraulic retention time (HRT). However, a drastic reduction of the biogas was observed in the system when the OLR was further increased from 6 to 7 gCOD/L. day. The reduction of biogas production in the system was due to the shock load received over the degradation capacity of the microbial population (Oliveira and Doelle 2015; Bong et al., 2017; Latha et al., 2019).

Figure 3B demonstrates the methane composition produced at different OLRs. The percentages (%) of methane composition in the CSTR were 72, 68, 63, 85, 42 42, and 36 at OLRs 1.3, 2, 4, 6, 7, and 8 gCOD/L. day, respectively. A sharp inclination on methane composition at OLR 6 gCOD/L. day with 85% of methane gas can be observed in Figure 3B. Then, with a massive drop in the methane composition once the OLR reached 7 and 8 gCOD/L. day, it can be deduced that the maximum OLR for continuous AD for the CMFW was at 6 gCOD/L. day. An OLR higher than 6 gCOD/L. day will shock load the system, leading to the loss of methanogens (Oliveira and Doelle 2015; Bong et al., 2017; Latha et al., 2019). Moreover, the shock load will cause a high layer of scum formation, which entraps the gas bubbles, hindering the transport of gas bubbles to the headspace (Paudel et al., 2017; Tyagi et al., 2018).

The effect of different OLRs 1.3 to 8 gCOD/L. day for the specific biogas production (SBP) and specific methane production (SMP) in the CSTR is shown in Figure 3C. It was observed that the SBP and SMP decreased with the increased OLR. The highest CMFW, SBP, and SMP were 2.46 L biogas/gCOD fed and SMP 1.77 CH_4_/gCOD fed at OLR 1.3 gCOD/L. day. Furthermore, the OLR 6 gCOD/L. day can be depicted as the maximum OLR range for CMFW digestion. The SBP and SMP drop instantaneously as it reaches the OLR 7 gCOD/L. day. It turns out the lowest SBP and SMP being monitored was 0.03 L biogas/gCOD fed and 0.01 CH_4_/gCOD at the OLR 8 gCOD/L. day. Optimum digestion and methane production can occur if the amount of bacteria present is balanced with the available and essential nutrients in the digester (Matheri et al., 2017). A high OLR creates a souring effect on the digester due to the accumulation of VFAs, thus leading to an overall decrease in the biogas production, percentage of methane, and the SMP and SBP.

Figure 3D shows the biogas and methane production rate of the CMFW in the CSTR with a progressive inclination of OLR phases. The gas production rate (GPR) and methane production rate (MPR) increased as the OLR increased higher from 1.3 gCOD/L. day to 4.0 gCOD/L. day. At the OLR 6.0 gCOD/L. day, the GPR and MPR were dynamically increased with the maximum GPR and MPR at 8-L biogas/L digester. day and 6.8-L CH_4_/L digester. day. A significant drop in the GPR and MPR was observed once the CMFW OLR reached 7.0 gCOD/L. day. While the SBP and SMP will indicate the feedstock digestibility’s efficiency to turn organic into biogas and methane, the GPR and MPR reflect the biogas and methane yield produced. Micolucci et al.’s study on the pilot scale comparison for single- and two-stage thermophilic anaerobic digestion of food waste resulted in the GPR 7 m^3^ biogas/m^3^ digester. day when the OLR is 7 kg/m^3^ day (Micolucci et al., 2018). Valentino et al.’s study on the development of pilot AD for urban bio-waste, a mixture of the OFMSW and waste-activated sludge resulted in the GPR 1.38 m^3^ biogas/m^3^ digester. day at the OLR 2.5 kg/m^3^. day (Valentino et al., 2019).

#### 3.4.2 Commercial Malaysian Food Waste Organic Removal Efficiency in a Continuous Stirred-Tank Reactor

**FIGURE 4:**
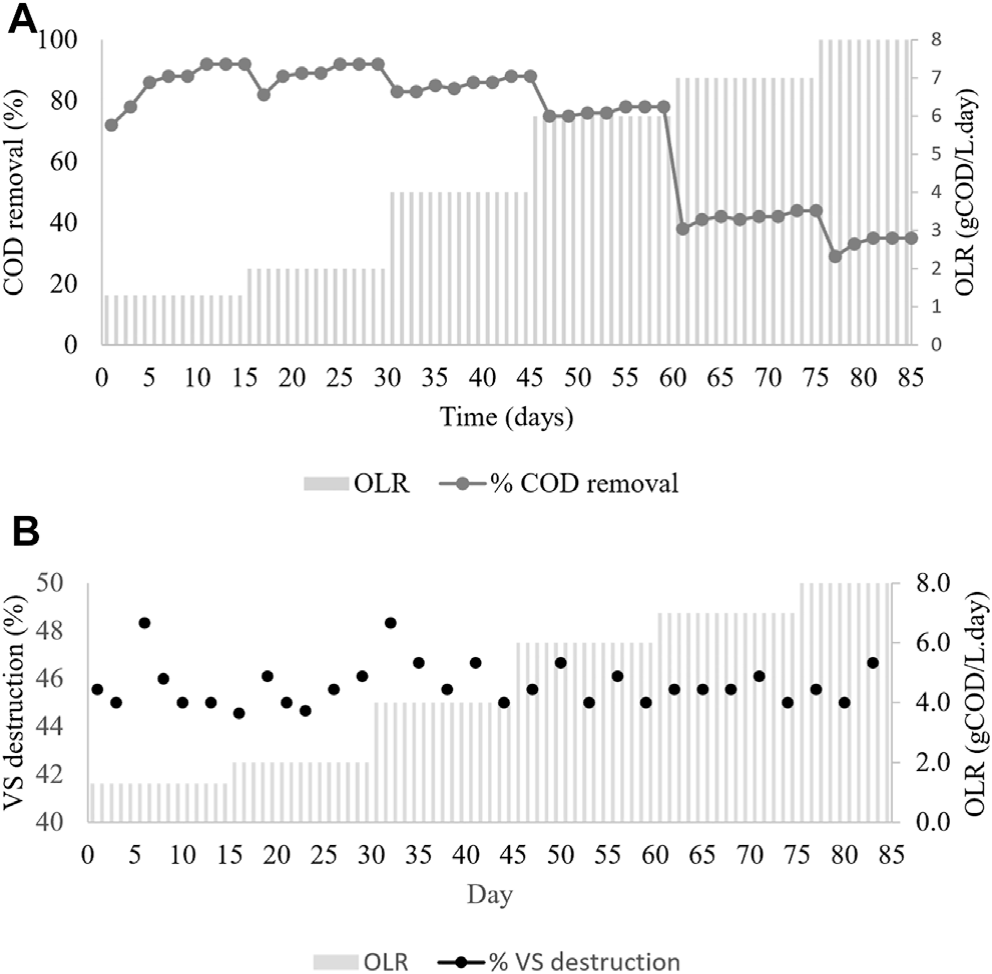
CMFW organic removal efficiency in CSTR. **(A)** COD removal **(B)** Volatile Solid destruction.

Figure 4A shows the changes in the OLR corresponding to the COD removal efficiency in the CSTR during the period of the operation. Although in terms of volatile solid destruction of the CMFW maintained more than 45% removal, as shown in Figure 4B, it was observed that at the lower OLR 1.3 and 2 gCOD/L. days, around 92% of COD removal was removed and recorded at a stable state. Further increment of the OLR up to 4.0 and 6.0 gCOD/L. day had reduced the COD removal efficiency to 88–78%. Once the OLR was at 6 gCOD/L. day, which was stable, the COD concentration was further increased to 7 gCOD/L. day. However, an increase in the COD concentration drastically reduced the COD removal efficiency to approximately 41% and decreased to 35% at the OLR 8 gCOD/L. day.

A significantly decreased COD removal at 7 and 8 gCOD/L. day was attributed to the overfed of the digester and resulted in microbial population inhibition and could not withstand the changes. At OLR 7.0 and 8.0 gCOD/L. day, valeric acid starts to accumulate in the system at 143.30, 746.27 mg/L, respectively, as shown in Table 6. Moreover, the total VFA in the system severely increased up to 1480.52, 2378.87 mg/L. This is in line with the study by Pramanik et al. that stated that volatile fatty acid accumulation leads to inhibitory substrates and causes an acidic condition in the digester. As the inhibitory substrate is beyond microbial capacity, low efficiency in COD removal can be seen, leading to a system failure (Pramanik et al., 2019).

**TABLE 6:** Volatile fatty acids.

OLR (gCOD/L.day)	Acetic Acid (mg/L)	Propionic Acid (mg/L)	Butyric Acid (mg/L)	Valeric Acid (mg/L)	Total VFA (mg/L)
1.3	265.05	118.35	146.49	ND	529.89
2.0	453.37	71.74	370.09	ND	895.20
4.0	269.50	293.28	789.03	ND	1351.81
6.0	264.80	197.01	474.22	ND	936.02
7.0	336.91	106.68	893.62	143.30	1480.52
8.0	505.98	551.31	575.3	746.27	2378.87

ND: Non-Detected.

#### 3.4.3 Continuous Stirred-Tank Reactor Digester Stability

**FIGURE 5:**
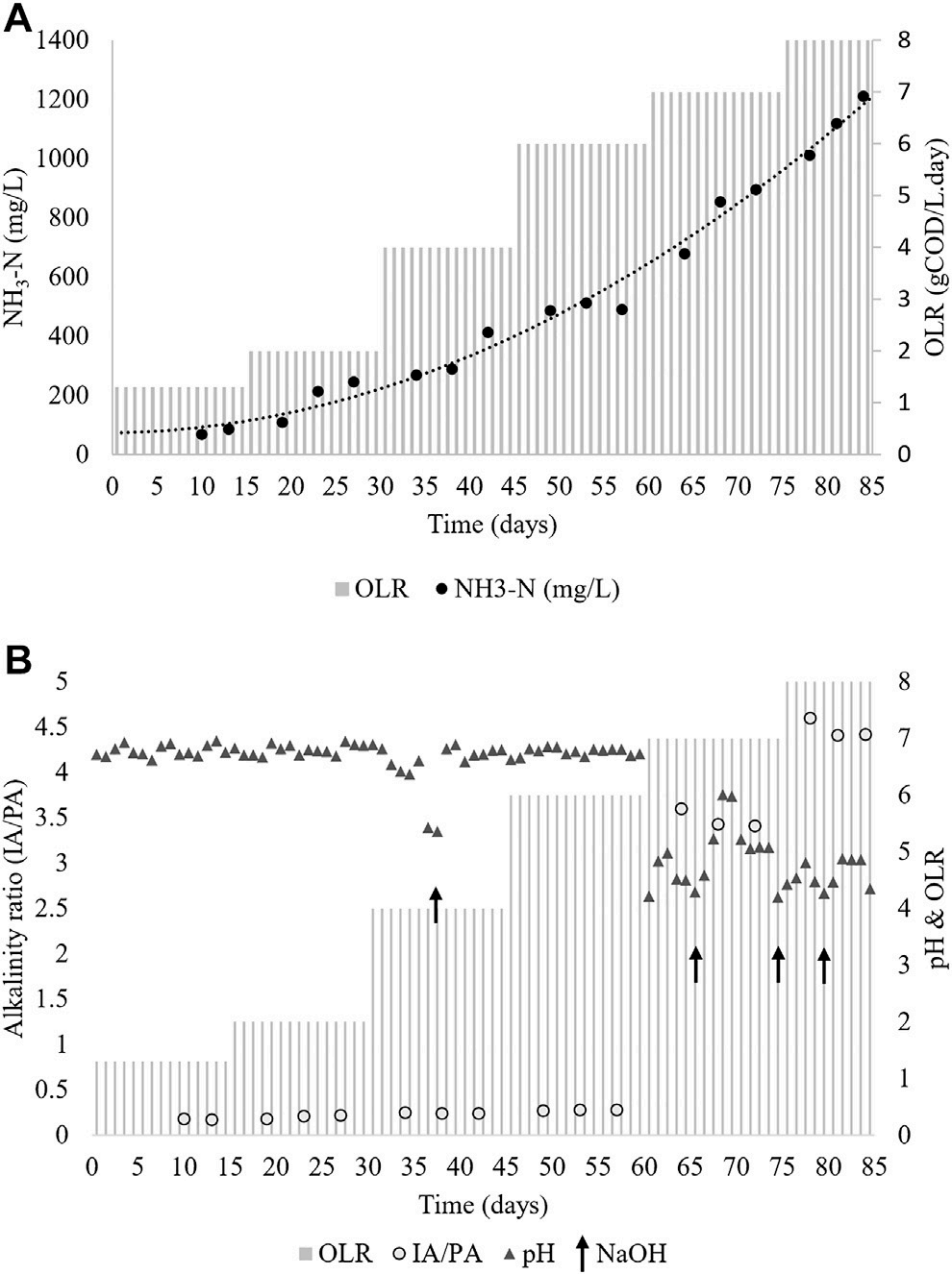
CSTR digester stability. **(A)** Ammonia nitrogen **(B)** pH and alkalinity ratio.

Figure 5A shows the NH_3_-N level in the effluent of the CMFW digestion process. From the observation, an increasing trend of NH_3_-N values is corresponding to the increase in COD levels and organic loading rates. Interestingly, although the level of ammonia increased, it neither inhibited nor caused any significant changes in the reactor operation, especially at OLRs 1.3, 2, 4, and 6 gCOD/L. day was observed.

The result of NH_3_-N in the CSTR, shown in Figure 5A, is comparable with the BMP test results, as presented in Table 5. During the OLR 2 gCOD/L. day, the NH_3_-N concentration was at 245 mg/L, whereas during the BMP test, the samples with OLRs 1.92 and 3.83 gCOD/L. day resulted in an NH_3_-N concentration of 187.5 mg/L and 272.5 mg/L. Corresponding to the increase of OLR 7 and 8 g/L, the digestion process was inhibited by the souring of the system due to excessive accumulation of VFAs (Table 6). Also, the NH_3_-N concentration was increased as well at this stage. This scenario may be attributed to the changes in the dynamics of mesophiles, whereby the higher activity of acidogens and the lower activity of methanogens are due to the overload of organics (Khanal 2008). The hydrolytic stage is often considered the first stage of biodegradation of particulate organic matter in which waste is broken down into simple sugars and VFAs (Morosini et al., 2016; Peng et al., 2018), although with lower pH acidogens which can produce acidic intermediates, leading to the accumulation of VFAs.

In contrast, methanogens are sensitive with a narrow pH range; the process of VFA consumption could be quickly ceased when pH levels drop (Ye et al., 2018). Furthermore, excess free ammonia blocked acetate metabolism, which was the origin of the process instability. Accumulated acetate caused feedback inhibition at the acetogenesis stage, resulting in the accumulation of other long-chain VFAs. VFA accumulation then reduced the abundance of acetogens, which upset the balance of microbial degradation networks, thus leading to the process of instability in the digester (Peng et al., 2018).

The digester stability is essential for the optimal microbial activity during the digestion process. Figure 5B shows the pH profile of the CSTR. The pH was stable during the steady-state of the OLR 1.3–6 gCOD/L. day where the pH was at 6–7 except when the OLR reached 7 gCOD/L. day onward. To ensure process stability in this study, NaOH was used as the buffer when the pH drops. It can be seen at the OLR 4 gCOD/L. day, a shot of NaOH aided in stabilizing the digester. However, the addition of NaOH slightly and temporarily improved the digester performance at OLR 7 and 8 gCOD/L. day before the system led to a worsening of the digester instability. As aforementioned in the previous discussion, the digester instability is due to the overfeeding of the organic into the system. Subsequently, it changes the mesophile dynamic in the system. Fermentative bacteria can withstand a wide range of pH between 4.0 and 8.5; thus, the digestion process was performed although the OLR was reaching 7 gCOD/L. day (Zhang et al., 2014). However, methanogens are sensitive to pH where their working pH range is between 6.5 and 8.0 (Zhang et al., 2014; Mirmohamadsadeghi et al., 2019), while its optimum is at pH 6.5–7.2 (Zhang et al., 2014). Hypothetically, it implies that methanogens and syntrophic bacteria were overwhelmed with VFAs produced by fermentative bacteria, and this organism is unable to keep up with the high production of fermentation products (Padmasiri et al., 2007). Consequently, the pH drop inhibits the syntrophic bacteria, acetogen, and methanogen performance. Thus, it leads to an unstable digestion system and subsequently resulted in a significant pH drop.

Changes in the pH of the digester during the operation are related to its buffering capacity, which can be monitored through the alkalinity ratio of the system. Figure 5B shows the alkalinity profile of the CSTR during the period of the operation at various OLRs. The maximum alkalinity ratios attained at OLR 1.3, 2, 4, and 6 gCOD/L. day were 0.187, 0.22, 0.24, and 0.28, which depicts a stable operating performance. However, a massive increase in the value of alkalinity up to 3.4 and 4.4, which occurred at OLR 7 and 8 gCOD/L. day, indicates inhibition of the digester due to shock loading. The high protein and lipid contents of food waste make it a challenging material for anaerobic digestion due to the inhibition caused during degradation. The functional anaerobic digestion system depends on the buffering capacity and the degree of adaptation of the microorganisms (Capson-Tojo et al., 2017). Alkalinity is an important parameter in anaerobic digestion that shows the capability of a solution to withstand a drop in pH produced by the release of organic acids. It is also termed as the system buffer capacity (Wong et al., 2009). The ability of methanogenic bacteria to resist higher VFA accumulation is highly dependent on the alkalinity value of the system and its buffering capacity. Thus, methane production processes in a bioreactor are a function of alkalinity and pH stability (Bong et al., 2017). The desirable alkalinity in the bioreactor is between 2000 and 4,000 mg CaCO_3_/L, and the alkalinity ratio should be less than 0.3 (Romero-Güiza et al., 2016).

### CMFW Biogas Kinetic Study

This study used a mathematical model using the modified Gompertz model to fit the biogas production. This kinetic study was performed using the data obtained from the BMP test. The biogas yield potential, P, maximum biogas potential production rate, R_m_, lag phase, λ, and its root-squared value, *R* ^2^, were determined using the solver analysis tools in Microsoft Excel 2016. Table 7 shows that all the samples are fitted with the model. The modified Gompertz model showed a satisfied root squared, *R* ^2^, at the range of 0.9832–0.9983. Overall, the biogas yield potential, P (0.345–2.618 L), and the maximum biogas potential production rate, R_m_ (0.0218–0.1573 L/day), were increased as the OLR increased and small ratio of the inoculum toward feed. In this study, the controlled CMFW mesophilic anaerobic digestion lag phase for the process was 0.2 days for all samples.

**TABLE 7:** Modified Gompertz kinetic model of CMFW biogas production.

Sample	I/F	OLR	P [L]	R_m_ [L/day]	λ [day]	*R* ^2^
Blank	−	−	0.345	0.0218	0.2	0.9904
*A*	300:1	0.38	0.395	0.0241	0.2	0.9974
*B*	150:1	0.77	0.521	0.0364	0.2	0.9897
*C*	100:1	1.15	0.606	0.0430	0.2	0.9832
*D*	75:1	1.53	0.677	0.0434	0.2	0.9877
*E*	60:1	1.92	0.755	0.0628	0.2	0.9824
*F*	30:1	3.82	2.618	0.1573	0.2	0.9983

Furthermore, the model demonstrated that the CMFW substrates had a higher biogas potential than a recent study (Khadka et al., 2022). The authors obtained the value of R_m_ as 0.0252 L/day. Thus, this reaffirmed the variation of food waste quality leads to wide-ranging biogas production rate outcomes. Likewise, this study is comparable with that of Jaman et al., which obtained a modified Gompertz model with *R* ^2^ at 0.9208 and R_m_ 0.1 L/day when using local food waste in Malaysia. Moreover, the study found that the modified Gompertz model for biogas production was fitted with food waste co-digested with the chicken manure. However, the chicken manure substrate fits well with a logistic model (Jaman et al., 2022).

On the other hand, the modified Gompertz model has been used to describe the co-digestion process of food waste, sewage sludge, and glycerol (Silva et al., 2018). Significantly, the study determined that the co-digestion of food waste and sewage sludge had resulted in P, R_m_, *λ*, and *R* ^2^ of 0.2917 L, 0.0257 L/day, 0.15 days, and 0.9891, respectively. Also, this model has been applied to describe biogas production in mesophilic anaerobic food digestion with a focus on the effect of waste cooking oil in school canteen food waste (Li Y. et al., 2018). The study determined that the model had a good fitting with all samples with an acceptable *R* ^2^ at a range of 0.9173–0.9822.

## 4 Conclusion

In this study, the CMFW characteristic has been established. It was found that the CMFW is significantly high in organic at 230 g COD/L and acidic (pH 4.5) with a carbon to nitrogen ratio of 121:1. The evaluation of the CMFW digestion performance in a batch process during the BMP test showed that the maximum methane composition was up to 81%, with the maximum organic removal efficiency at a range of 98%. In terms of biogas productivity, during the BMP test, it was found that biogas production was well-fitted to the modified Gompertz model with *R* ^2^ at 0.9983 and maximum biogas potential production rate, R_m_ 0.1573 L/day. The maximum CMFW SMP during the BMP test was at 0.952 L. CH_4_/L.COD fed was obtained at 3.83 gCOD/L. day OLR whereas the SMP in the CSTR with 1.77 L. CH_4_/L.COD fed was obtained at 1.3 gCOD/L. day OLR. The CSTR process showed a good stability condition as pH was well-maintained at pH 6.6–6.7 and an alkalinity ratio of 0.28. Overall, the analyzed performance parameter during the BMP test and the continuous digestion process indicated that the CMFW responds well with the anaerobic digestion treatment. Thus, an attempt to step up further using the CMFW substrate for OFMSW AD is positive, and the scale-up analysis in the pilot system is recommended as a basis for future commercial plant development.

## Data Availability

The original contributions presented in the study are included in the article/Supplementary Material, further inquiries can be directed to the corresponding author.
